# Polymers and the Environment: Some Current Feature Trends

**DOI:** 10.3390/polym15092093

**Published:** 2023-04-27

**Authors:** Jesús-María García-Martínez, Emilia P. Collar

**Affiliations:** Polymer Engineering Group (GIP), Polymer Science and Technology Institute (ICTP), Spanish National Research Council (CSIC), C/Juan de la Cierva, 3, 28006 Madrid, Spain

In the early 1980s, the first global environmental crisis occurred with an emphasis on the role of plastics in big cities’ massive solid waste streams. It was apparent then (and now) that the best environmental management practices required solid scientific and technical knowledge (usually under technical standards). Once at the end of their useful life, these plastics become involved in their materials (polymers and additives) into a circular economy strategy conjugated with the non-steady too scenarios of the other key sectors of the economy, industry, society, and policy. Thus, a twofold perspective, applied and academic, to link tandem polymers and the environment has led, forty years later, to a wide polymer research field devoted to continuously improving the environmental performance of polymer and polymer-based materials. This strategy comprises all the steps in the polymer management chain, from the raw materials to the polymers themselves, many of which come from classical and/or renewable sources (the so-called bioplastics). There is a need to improve the processability, ultimate properties, and performance through friendly environment additives; the recyclability of the materials; and innovative processes that will allow for better mechanical and/or energy recovery, including chemical recycling. Therefore, this Special Issue includes a number of interesting works related to this frontrunner polymer R&D area [1,2]. The articles compiled in this volume fully match all the philosophies mentioned above. 

Torre, Puglia, and Luzi have contributed a fascinating review paper on the preparation and applications of green thermoplastic and thermosetting nanocomposites based on nanolignin [3]. In this review article, the authors pay attention to lignin’s valorization from plenty of lignocellulosic wastes. Lignin is a natural polymer with a cross-linked structure, valuable antiradical activity, and unique thermal and UV absorption properties besides being biodegradable, making it very valuable to be used either as a nanofiller or raw material to synthesize eco-friendly polymeric matrices. In the resume, this interesting review summarizes frontrunner synthesis methods for bio-based and/or biodegradable thermoplastic and thermosetting nanocomposites jointly with applications of lignin nanoparticles as reinforcements in nanocomposites. 

The other review in this volume, authored by Infurna, Caruso, and Dintcheva [4], is devoted to using biochar particles as reinforcement in sustainable materials. The article is focused on how the conversion of polymer waste, food waste, and biomasses let obtain a solid phase named char/biochar particles (in addition to fuels and syngas) to be used in a wide variety of eco-friendly applications based on its chemical composition, porosity, and absorption ability depending of the thermochemical decomposition method used. Furthermore, the authors mention the actual uses of these particles as fertilizers for soil retirement and water treatment, introducing new services for formulating sustainable polymer and biopolymer-based composites, or as fillers in asphalts. Finally, the authors describe the advantages and disadvantages of using these biochar particles in polymer composites. 

The article by Farroni et al. [5] is devoted to the fractional calculus approach to reproduce the material viscoelastic behavior based on an exceptionally reliable model parameterization procedure using the poles–zeros formulation. In the article, the authors state that the design of modern products and processes cannot leave out the usage of viscoelastic materials that provide extreme design freedoms at relatively low cost, paying attention to the possibility of reuse and recycling of these types of materials. The authors conclude that a limited number of the experimental curves could feed the identification methodology and predict the complete viscoelastic material behavior.

Based on the case study of a recycled opaque polyethylene terephthalate (r-O-PET), Arbiter, Santana, Maspoch, et al. report the implications of the circular economy in the context of plastic recycling [6]. The authors have evaluated r-O-PET with TiO_2_ as reinforcement for recycled polypropylene matrices (r-PP), applying the life cycle assessment to different scenarios. By considering two different recycled blends and two virgin raw plastics as reference materials, when comparing the environmental performance of the proposed treatments, they found a significant reduction of environmental costs when substituting virgin materials PP and PA66 for the blends evaluated in this study.

Perše et al. contributed to this issue with a valuable study on the influence of stabilizers on the rheological, thermal, and mechanical properties of recycled polypropylene [7]. In their article, the authors have shown that after twenty recycling steps by successive reprocessing of polypropylene, the well-known fact that the main degradation processes of polypropylene are based on oxidation and chain scission, which the addition of such stabilizer can efficiently delay. Hence, they find that a tiny proportion of the additive dramatically improves the properties of the reprocessed polypropylene at least after twenty reprocessing rounds, acting as a hardener by promoting cross-linking reactions between polymer chains. 

Dipa et al. investigated the reuse of packaging single-use plastic wastes recovered from landfill in the current context of a circular economy [8]. In essence, the authors study LDPE-based packaging films mostly end up in landfill after single-use because they are not commonly recycled despite their flexible nature, low strength, and low cost due to the expensive step of separation and sorting the different plastic waste streams that limit a broader use in recycling routes. Their research investigates the properties of PE-based waste mixed plastics with traces of PP, concluding that the final properties and absence of degradation provide a scientific insight into adopting these materials to return to the material markets. 

An article on optimizing the polymer costs and efficiency in alkali–polymer oilfield applications is the proposal published by Hincapie et al. [9]. Indeed, the authors have performed a fascinating study by presenting various evaluations critical to prior field applications. For this purpose, the authors combine different laboratory approaches to optimize the usage of polymers in combination with alkalis to improve project economics. In essence, the authors optimize the performance of alkali–polymer floods by employing lower polymer viscosities during the injection but increasing polymer viscosities in the reservoir owing to the “thermal aging” of the polymers at a high pH. They evaluated alkali–polymers in the 8-Torton Horizon reservoir of the Matzen field in Austria in the Schoenkirchen area. Finally, the authors conclude that alkali–polymer injection leads to substantial incremental oil production of reactive oils. Therefore, alkali–polymer flood displacement efficiency must be evaluated by incorporating the aging of the polymer solutions, reaching significant cost savings in the process.

Arrieta et al. use yerba mate waste to obtain a kombucha beverage that produces microbial cellulose as a helpful byproduct to reinforce a mechanically recycled poly(lactic acid) (r-PLA) matrix [10] in their article titled Plasticized Mechanical Recycled PLA Films Reinforced with Microbial Cellulose Particles Obtained from Kombucha Fermented in Yerba Mate Waste. The authors also use microbial cellulosic particles obtained from the fresh yerba mate for comparison. To simulate the revalorization of the industrial PLA products rejected during the production line, PLA was subjected to three extrusion cycles and further addition of plasticizer. The transparent films showed flexible and good structural and mechanical performance, jointly with antioxidant properties revealed by contact with fatty food models, implying the potential of this recycled food waste in food packaging and agricultural films.

Considering that plastic particles are widespread in the environment, including terrestrial ecosystems, and they may disturb the physicochemical properties of soil and plant growth, Ewa Liwarska-Bizukojc has studied the effect of different biobased polymers on vegetal plants evolution [11]. In her investigation, she considered five innovative bio-based plastics of varying chemical composition and application on the early growth of higher plants (sorghum, cress, and mustard), being found that at the early stages, the growth of monocotyledonous plants seemed not to be affected at all by the presence of each bioplastic studied. The plastics studied were based on polylactic acid (PLA), poly-hydroxy–butyrate–valerate (PHBV), and polybutylene succinate (PBS). Simultaneously, she found that some bio-based plastics inhibited the root growth and stimulation of shoot growth of dicotyledonous plants. 

Finally, Dorigato, Morreale, and coworkers present an evaluation of the physical and shape memory properties of fully biodegradable poly-lactic acid (PLA)/poly-butylene adipate terephthalate (PBAT) blends, taking in mind that this type of materials in combination has potential application in the packaging field [12]. One study aim is to provide information about the thermomechanical behavior of PBAT poorly characterized in the literature, mainly concerning the effect of the addition of PBTA on the shape memory properties of PLA. A microstructural investigation was conducted, evidencing the immiscibility and the low interfacial adhesion between the PLA and PBAT phases. The authors conclude the presence of PBAT hinders the shape memory of PLA and explain this fact in terms of the incompatibility of both polymers. Further, the authors claim that their future works will explore the shape memory effect of the samples once compatibilized with suitable additives.

To conclude, as the editors, we can say that the topic “Polymers and the Environment” arises as an essential framework in the field of Polymer Science and Technology in the present and near future. For this reason, a second Special Issue on the topic, which will be published by *POLYMERS* in 2023, is in progress, and new contributions will be welcomed. 
